# HuD Interacts with *Bdnf* mRNA and Is Essential for Activity-Induced BDNF Synthesis in Dendrites

**DOI:** 10.1371/journal.pone.0117264

**Published:** 2015-02-18

**Authors:** Filip Vanevski, Baoji Xu

**Affiliations:** Department of Pharmacology and Physiology, Georgetown University Medical Center, Washington, D.C., United States of America; Institut National de la Santé et de la Recherche Médicale (INSERM U901), FRANCE

## Abstract

Highly specific activity-dependent neuronal responses are necessary for modulating synapses to facilitate learning and memory. We present evidence linking a number of important processes involved in regulating synaptic plasticity, suggesting a mechanistic pathway whereby activity-dependent signaling, likely through protein kinase C (PKC)-mediated phosphorylation of HuD, can relieve basal repression of *Bdnf* mRNA translation in dendrites, allowing for increased TrkB signaling and synaptic remodeling. We demonstrate that the neuronal ELAV family of RNA binding proteins associates *in vivo* with several *Bdnf* mRNA isoforms present in the adult brain in an activity-dependent manner, and that one member, HuD, interacts directly with sequences in the long *Bdnf* 3' untranslated region (3'UTR) and co-localizes with *Bdnf* mRNA in dendrites of hippocampal neurons. Activation of PKC leads to increased dendritic translation of mRNAs containing the long *Bdnf* 3'UTR, a process that is dependent on the presence of HuD and its phosphorylation at threonine residues 149 and/or 165. Thus, we found a direct effect of HuD on regulating translation of dendritic *Bdnf* mRNAs to mediate local and activity-dependent increases in dendritic BDNF synthesis.

## Introduction

Brain-derived neurotrophic factor (BDNF) is an important molecule involved in learning and memory in the adult brain, with demonstrated roles in regulating synaptic plasticity [1–3]. The mechanisms by which BDNF regulates synaptic strength have become increasingly well understood. BDNF stored in dendritic vesicles is released both constitutively and through an activity-dependent pathway. Released BDNF can potentially bind to TrkB receptors on both pre- and post-synaptic terminals, leading to retrograde and anterograde signaling [4–7]. Recent studies highlight the significance of regulation of local translation of mRNAs in activated spines as one way in which BDNF can specifically modify synapses in response to stimulation [8–11]. Despite the increasing understanding of how a number of important genes are regulated by BDNF signaling to modify the structure and composition of synapses, less is known about how BDNF itself is regulated. While recent work has begun to show some of the key mechanisms at play in regulating dendritic BDNF synthesis [12], there is still much to be determined about the various pathways involved.

Our previous studies have shown that sequences in the long *Bdnf* 3' untranslated region (3' UTR) are sufficient to traffic mRNAs to distal dendrites, and that *Bdnf* mRNAs lacking the long 3' UTR are primarily restricted to the soma [11], suggesting the *cis*-acting elements regulating dendritic *Bdnf* mRNA translation are located in this region. Indeed, it has been found that under basal conditions the long *Bdnf* 3' UTR is a repressor of translation, and neuronal activity can increase the translation of mRNAs containing this sequence [13]. The *trans*-acting factors binding to *Bdnf* mRNAs to regulate their local translation, however, are not known. The neuronal Hu proteins, a family of RNA binding proteins (RBPs), are ideal candidates. They are RBPs that stabilize their target mRNAs, can lead to increased translation in response to neuronal activation, and have been implicated in regulating learning and memory processes [14–16]. One of these proteins, HuD, has been the focus of a number of studies [17]. Protein kinase C (PKC) activity is necessary for phosphorylation on HuD to mediate its associated increases in target mRNA stability and translation [18,19]. It has been shown that PKC-dependent phosphorylation of HuD and its association with the short *Bdnf* 3' UTR are important for upregulation of BDNF translation and BDNF-dependent long-term potentiation (LTP) [19,20]. However, these studies did not examine the interaction of HuD with the long *Bdnf* 3' UTR.

Here we present *in vitro* and *in vivo* findings that bring to light a possible mechanism for linking neuronal activation to increased local protein synthesis of dendritically localized mRNAs. Immunoprecipitations of messenger ribonucleoprotein (mRNP) complexes from mice under basal or stimulated conditions reveal an activity-dependent association of *Bdnf* mRNAs with neuronal Hu proteins. Observations of dissociated rat hippocampal cultures manipulated genetically and pharmacologically show the importance of HuD and PKC in regulating the dendritic translation of mRNAs containing *Bdnf* 3' UTR sequences, and RNA electrophoretic mobility shift assays (REMSAs) point to a direct interaction between the HuD protein and *Bdnf* mRNA sequences specific to the long 3' UTR.

## Materials and Methods

### Animals and DNA constructs

Pregnant female Sprague Dawley rats were purchased from the Charles River Laboratories (Wilmington, MA, USA). Both genders of mice were used in this study. The Georgetown University Animal Care and Use Committee approved the animal procedures performed in this study.

The human synapsin (hSYN) promoter was amplified from the lentiviral FSW plasmid, introducing a 5' BglII site and a 3' XhoI site, allowing insertion between these sites into the pcDNA3.1(-)/*myc*-His A vector (Invitrogen Corporation, Carlsbad, CA, USA), swapping out the cytomegalovirus (CMV) promoter and generating pSYN-cDNA3.1. A DNA fragment encoding the Src tyrosine kinase myristoylation sequence (MGSSKSKPK), atggggagtagcaagagcaagcctaag, was added to the 5'-end of a PCR-amplified EGFP insert from plasmid pd1EGFP-N1 (Clontech, Mountain View, CA, USA), which was cloned into pSYN-cDNA3.1 between the BamHI and NotI sites, generating pSYN-mdGFP. A DNA fragment encoding the SV40 large T antigen nuclear localization sequence (PKKKRKV), ccaaagaagaagagaaaggtt, was added to the 3'-end of a PCR-amplified EGFP insert from plasmid pSYN-mdGFP, which was cloned back into the plasmid to generate pSYN-mdGFPn. The mouse genomic sequences for the two *Bdnf* 3' UTRs, A (short) and A*B (long) where the polyadenylation signal AATAAA for the first polyadenylation site was changed to TTTTTT, were derived as previously described [11] and cloned into pSYN-mdGFPn between the NotI and AflII sites, generating pSYN-mdGFPn-A and pSYN-mdGFPn-A*B. DNA sequences complementary to the 5' UTRs of *Bdnf* mRNA isoforms I, IIc, IV and VI were PCR amplified from mouse brain cDNAs, introducing a 5' XhoI site and a 3' EcoRI site, allowing for cloning into pSYN-mdGFPn-A*B between these sites. The following primers were used: f-exI (ctctctcgagtaaagcagtagccggctggt); r-exI (ctctgaattctgtggctttgctgtcctgga); f-exIIc (ctctctcgaggctttggcaaagccatccac); f-exIV (ctctctcgagacccactttcccattcaccg); f-exVI (ctctctcgagccaatcgaagctcaaccgaa); r-IIc,IV,VI (ctctgaattccactcttctcacctggtgga).

HuD shRNA knockdown constructs were generating by annealing the following oligonucleotides and inserting the dsDNA between the BglII and HindIII sites of pSuper (Oligoengine, Seattle, WA, USA), following the manufacturer's protocol: shHuD-f (gatcccccgtccgagctcggcctcaattcaagagattgaggccgagctcggacgtttttggaaa); shHuD-r (agcttttccaaaaacgtccgagctcggcctcaattctcttgaattgaggccgagctcggacgggg). The entire mouse HuD coding region was PCR amplified from mouse brain cDNA, introducing a 5' BamHI site and a 3' NotI site, cloning between these sites into pcDNA3.1(-)/*myc*-His A to generate pcDNA3.1-mHuD. Amino acids 149 and 165 of mouse HuD were found to be the highest ranked potential threonine targets for PKC phosphorylation using NetPhos 2.0 *in silico* prediction (http://www.cbs.dtu.dk/services/NetPhos/), and were substituted with alanine residues in pcDNA3.1-mHuD using the QuickChange II Site-Directed Mutagenesis Kit (Agilent Technologies, Santa Clara, CA, USA), generating pcDNA3.1-mHuDpd. The EGFP coding region from pEGFP-N1 was PCR amplified, introducing a 5' XhoI site and a 3' BamHI site, and cloned between these sites in pcDNA3.1-mHuD and pcDNA3.1-mHuDpd. The hSYN promoter was then cloned into these two new constructs as described above, generating pSYN-GFP/HuD and pSYN-GFP/HuDpd. The sequences corresponding to HuD amino acids 3–385 from pcDNA3.1-mHuD and pcDNA3.1-mHuDpd were PCR amplified, introducing 5' and 3' BamHI sites for cloning into the overexpression vector pGEX-2T (GE Healthcare) to generate pGEX-2T/HuD and pGEX-2T/HuDpd, respectively.

To generate biotin-labeled and unlabeled sense riboprobes for REMSA experiments, sequences corresponding to the mouse *Bdnf* coding sequence and 3' UTR, as well as the mouse *Nova1* 3' UTR, were PCR amplified from mouse brain cDNA and cloned into the pBluescript II KS (-) plasmid (Stratagene, La Jolla, CA, USA). The following primers were used: f-CDS (tctgcgaattcatgaccatccttttccttac); r-CDS (ttgatctcgagctatcttccccttttaatgg); f-A (tctgcgaattctggatttatgttgtatagat); r-A (ttgatctcgagaatctgttttctgaaagagg); f-B1 (tctgcgaattctctttcagaaaacagattaa); r-B1(ttgatctcgagggccattcagtcctatttca); f-B2(tctgcgaattcctgcggaggctaagtggagc); r-B2(ttgatctcgagcactcctaagatgaagcgat); f-B3(tctgcgaattcgaaaggaaacagaagtggac); r-B3(ttgatctcgagtttgaaaatatatttaaaaa); f-Nova1A (tgatcgagctctgagtgtccccattatacgtcag); r-Nova1A(ctgcaggatccagaaactgcactggctgctagcg). To generate DIG-labeled antisense and sense riboprobes for fluorescent in situ hybridization, the mouse cDNA sequence for the *Bdnf* coding region (GenBank accession number NM_001048139, nucleotides 521–1270) and the EGFP coding region from pEGFP-N1 (nucleotides 679–1398) were amplified by PCR and cloned into pBluescript II KS (-).

### mRNP immunoprecipitation

mRNPs were isolated as previously described [21] with modifications, using forebrain tissue from male and female adult mice. For the activity-dependent assays, mice were pre-treated with intraperitoneal (IP) injection of either 5 mg/kg atropine methyl nitrate (Sigma-Aldrich, St. Louis, MO, USA) alone, or in combination with 30 mg/kg Ro-32–0432 (Enzo Life Sciences, Farmingdale, NY, USA) for the PKC inhibition condition, 30 min prior to IP injection of 400 mg/kg pilocarpine nitrate (MP Biomedicals, Solon, OH, USA). Mice were euthanized 30 min later. Isolated forebrain tissue was washed in ice-cold PBS, transferred to 1 ml RNP buffer (100 mM KCl, 5 mM MgCl_2_, 10 mM HEPES, 0.5% Nonidet P-40) supplemented with 200 U/ml RNasin (Promega, Madison, WI, USA), protease inhibitors (5 μg/ml aprotonin, 5 μg/ml leupeptin, 0.2 mM Na_3_VO_4_, 1 mM phenylmethylsulfonyl fluoride; all from Sigma-Aldrich) and 10 μM DTT, homogenized with 12 strokes in a dounce homogenizer and frozen at-70°C. Lysate was thawed and clarified, reserving 5% for input normalization, and 200 μl (~6 mg protein) was added to 200 μl protein A/G beads (Thermo Fisher Scientific, Waltham, MA, USA) pre-coated with 5 μg of mouse α-nELAV antibody (16A11, Life Technologies, Grand Island, NY, USA) or 5 μg of mouse IgG isotype control (Santa Cruz Biotechnology, Santa Cruz, CA, USA), incubating for 2 hours with rotation at room temperature. Beads were washed 4X with NT2 and 1X with freshly prepared 1 M urea, treated with 50 μg proteinase K (Roche Diagnostics, Indianapolis, IN, USA) for 30 min at 55°C, and RNA was isolated using TRIzol reagent (Life Technologies) following the manufacturer's protocol, adding 5 μg glycogen prior to isopropanol precipitation. Following DNase I digestion and RNA isolation using TRIzol reagent, cDNA was generated using SuperScriptII reverse transcriptase (Life Technologies) and oligo dT primers.

### Quantitative real-time PCR

Real-time PCR was performed using 50 cycles with FastStart Universal SYBR Green Master (ROX) (Roche Diagnostics) on the StepOne Real-Time PCR System (Applied Biosystems, Foster City, CA, USA) using oligonucleotide pairs designed on Primer Express 3.0 software (Applied Biosystems) to span introns. The primers used were: F-ExI (ACTGAGTCTCCAGGACAGCAAAG); F-ExIIc (GTGGTGTAAGCCGCAAAGAA); F-ExIV (CAGAGCAGCTGCCTTGATGTT); F-ExVI (CAGAAGCGTGACAACAATGTGA); R-BDNF (CCTTCATGCAACCGAAGTATGA); F-Rpl10a (GAAGAAGGTGCTGTGTTTGGC); R-Rpl10a (TCGGTCATCTTCACGTGGC). Reactions were carried out in triplicate for each sample. For the association assays ΔC_t_ was calculated by subtracting the C_t_ for *Rpl10a*, a housekeeping gene whose mRNA does not associate with HuD [22], and ΔΔC_t_ was calculated as the ΔC_t_ for the α-nELAV IP minus the ΔC_t_ for the IgG isotype control IP, with fold-difference determined by 2^-ΔΔCt^. For the seizure-induction assays ΔC_t_ was calculated by subtracting the C_t_ for the input (relative percent recovery), and ΔΔC_t_ was calculated as the ΔC_t_ for the pilocarpine treatment minus the ΔC_t_ for the PBS control treatment, with fold-difference determined by 2^-ΔΔCt^.

### Local translation reporter assays

Primary dissociated hippocampal cultures were established and transfected as described previously [23]. The cultures were transfected with the indicated plasmids at 14 days in vitro (DIV) and assays were performed 48 hours later at 16 DIV. Pre-warmed and CO_2_-equilibrated Neurobasal media was used for all media replacements. For KCl stimulation experiments, media was removed and replaced with Neurobasal containing 30 mM KCl for 5 min, which was then replaced with unsupplemented Neurobasal for an additional 55 min. For PMA stimulation experiments, media was replaced with Neurobasal containing 100 nM Phorbol 12-myristate 13 acetate (PMA) (Enzo Life Sciences, Farmingdale, NY, USA) for 1 hour. For the assays with PKC inhibition, media was replaced with Neurobasal containing 1 μM GF 109203X for 30 min prior to KCl or PMA treatment as above, with 1 μM GF 109203X included in the stimulation media. Cells were fixed and processed as described for in situ hybridization and immunocytochemistry. Neurons having pyramidal-like morphologies were selected, and apical dendrites were chosen for analysis based on structural properties. Images were acquired on a Nikon Eclipse E800 microscope using PictureFrame software (Optronics, Muskogee, OK, USA).

### Immunocytochemistry (ICC)

Cultured neurons were fixed with 4% paraformaldehyde, blocked in a blocking buffer (10% BSA, 0.1% Triton X-100 in PBS) for 1 hour at room temperature, and incubated with primary antibodies in a dilution buffer (1% BSA, 0.1% Triton X-100 in PBS) overnight at 4°C. Mouse monoclonal antibody to HuD (16C12, Santa Cruz Biotechnology) was used at a 1:500 dilution for somatic expression analysis and at a 1:250 dilution for dendritic expression analysis. Rabbit polyclonal antibody to GFP (Clontech) was used at a 1:2,000 dilution. After washes, the coverslips were incubated with the appropriate DyLight 488, 594 or 649 secondary antibody (Jackson ImmunoResearch Laboratories, West Grove, PA, USA) at a 1:1,000 dilution, washed, mounted with gelvatol and analyzed by fluorescence or confocal microscopy.

### Fluorescent *in situ* hybridization (FISH)

*In situ* hybridization of cultured neurons was performed as described [11] with modifications. Antisense and sense RNA probes were synthesized from linearized plasmids using T3 and T7 RNA polymerases (Promega, Madison, WI, USA) and DIG-labeled ribonucleotides. Probe concentrations were 500 ng/ml for *Bdnf* mRNA and 100 ng/ml for GFP mRNA. For combined FISH and ICC, rabbit polyclonal antibody to GFP (Clontech) was included at a 1:2,000 dilution after the hybridization step. Coverslips were washed four times for 10 min in TNT buffer (100 mM Tris-Cl, pH7.5, 150 mM NaCl, and 0.05% Tween 20), and were incubated with secondary antibody as described above for ICC and washed again with TNT buffer. Then, *in situ* signals were amplified with the TSA Plus Fluorescein System (PerkinElmer) according to the manufacturer's instructions. The coverslips were mounted onto slides with gelvatol and examined by fluorescence or confocal microscopy. *In situ* hybridization images from the antisense probe and its sense control probe were taken with the same settings. Green fluorescence from GFP in transfected neurons was not detectable after the hybridization and wash steps.

### Image analysis

All images were analyzed using NIH ImageJ software. Background was subtracted using a “rolling ball” algorithm. Dendritic processes were straightened and binned into 50 μm sections for ICC or 20 μm sections for combined FISH and ICC. The mean fluorescence for each bin was calculated as the mean of the brightest pixel of each line transecting the dendrite crosswise, emphasizing puncta representing areas of local synthesis. A minimum of 30 dendrites for each of 3 separate experiments were analyzed for each condition. Co-localization experiments were analyzed by an intensity correlation coefficient-based (ICCB) method using the JACoP plugin (http://rsbweb.nih.gov/ij/plugins/track/jacop.html) for ImageJ. Confocal images of *Bdnf* mRNA FISH and HuD ICC were thresholded to include most of the dendritic process, and Pearson's and Manders' coefficients were calculated.

### RNA Electrophoretic Mobility Shift Assays (REMSA)

REMSAs were carried out as previously described [22] with modifications. Sense riboprobes were synthesized from linearized plasmids using T7 RNA polymerases (Promega) and biotin-labeled or unlabeled ribonucleotides (Roche Diagnostics). *In vitro* transcription reactions were treated with 0.1% SDS and 50 μg proteinase K at 55°C for 30 min, diluted to 130 μl and treated with 15 μl ammonium acetate stop solution (5 M ammonium acetate, 100 mM EDTA), extracted with an equal volume of phenol/chloroform, isopropanol precipitated at-70°C for 20 min, washed with 75% ethanol, and resuspended in 20 μl DEPC-treated ddH_2_O. For standard REMSA assays 2 nM labeled riboprobes were incubated with 2 μM recombinant GST-HuD or GST-HuDpd in 10 μl binding buffer (150 mM NaCl, 20 mM Tris, 1.5 mM MgCl_2_, 0.5 mM DTT, 0.5 μg/μl tRNA) for 20 min at room temperature, and for competition assays 8 nM unlabeled riboprobes were incubated with protein as above for 15 min before adding 2 nM labeled *Nova1* A riboprobe and incubating an additional 20 min. Samples were run on a 5% polyacrylamide gel in TBE buffer and nucleic acids were transferred to positively-charged nylon membranes (Roche Diagnostics) followed by UV-crosslinking. Membranes were blocked in Odyssey blocking buffer (LI-COR Biosciences, Lincoln, NE, USA) + 1% SDS for 30 min, incubated with streptavidin DyLight 800 (Thermo Fisher Scientific) diluted 1:10,000 in Odyssey blocking buffer + 1% SDS for 30 min, washed 3X for 5 min with PBS + 0.1% Tween 20 followed by a final wash with PBS, then imaged using the Odyssey infrared imaging system (LI-COR Biosciences).

## Results

### Neuronal Hu proteins are associated with *Bdnf* mRNAs in the mouse forebrain

Mouse *Bdnf* mRNAs can have either a short (0.35 kb) or long (2.85 kb) 3' UTR, depending on which of two polyadenylation sites is used for processing [24]. The long *Bdnf* 3' UTR has several AU-rich elements that could serve as binding sites for proteins that modulate RNA metabolism and translation. The neuronal ELAV (nELAV) family of RNA-binding proteins (RBPs), consisting of HuB, HuC and HuD, has been previously demonstrated to interact with several mRNAs to regulate their expression [21,25,26], and putative binding sites for these proteins are present within the long *Bdnf* 3' UTR, making them potential candidates for the trans-acting factors regulating local BDNF synthesis.

**Figure 1:**
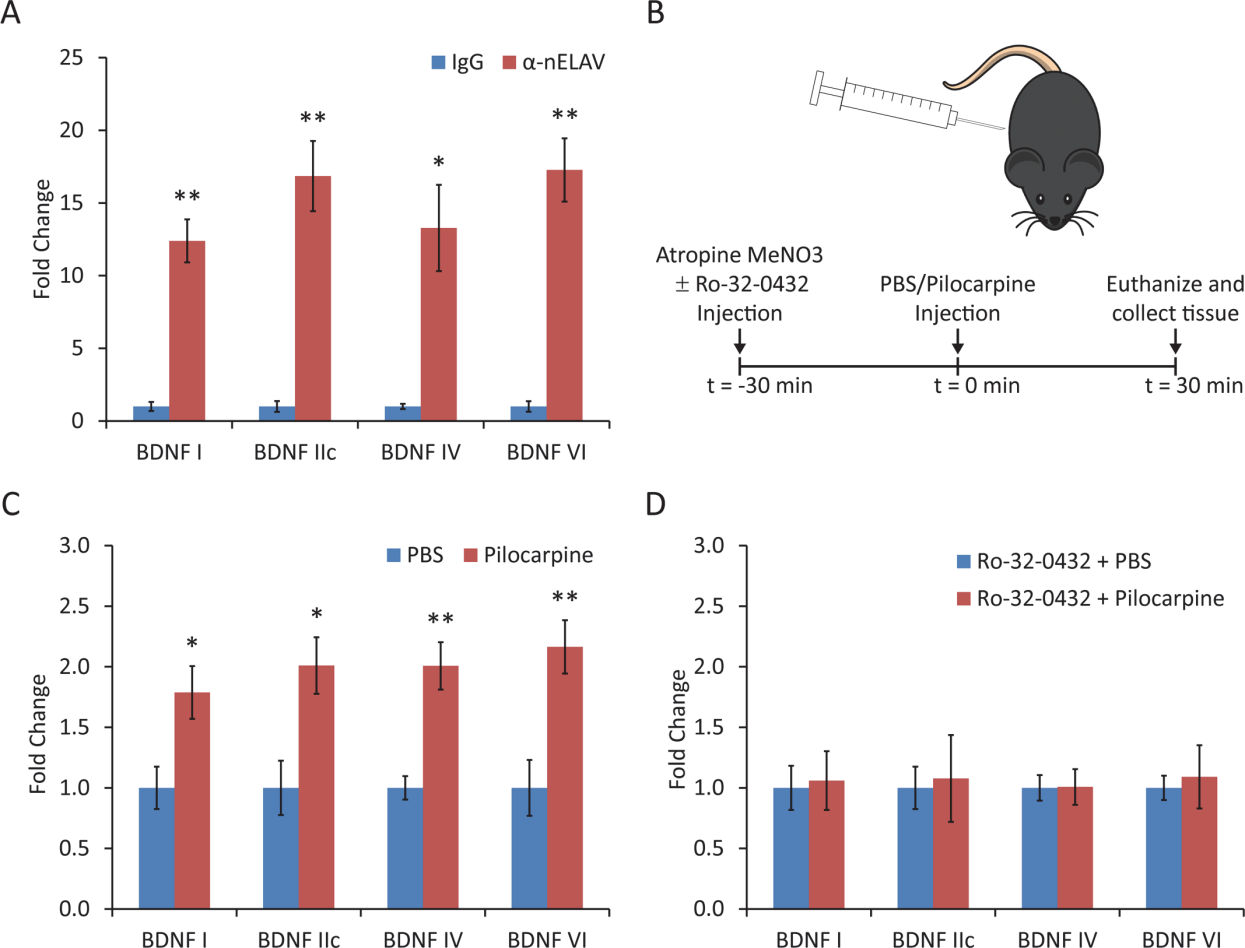
Quantitative RT-PCR analysis of *in vivo* mRNPs containing neuronal Hu proteins. (A) mRNPs were immunoprecipitated from mouse whole brain lysates using either the HuC/HuD neuronal protein antibody (16A11) or mouse IgG isotype control, and subjected to quantitative RT-PCR analysis using primers specific to *Bdnf* mRNA isoforms I, II, IV and VI, as well as primers for *Rpl10a* as reference control. Fold change was calculated as 2^(-ΔΔCt)^, using values from the mouse IgG IP as control (Mean ± SEM; n = 3; *, p<0.05; **, p<0.01; student's t-test). (B) Schematic showing the drug injection protocol for seizure induction. Atropine MeNO_3_ was injected alone or with Ro-32–0432 (systemic PKC inhibitor), followed 30 minutes later by injection of pilocarpine for seizure induction or PBS as a negative control, and mice were euthanized 30 minutes later, collecting forebrain tissue for isolation of mRNPs. (C) mRNPs were immunoprecipitated using lysates from mice injected with either pilocarpine or PBS. Percent recovery versus control was calculated as the ratio of the Ct for the IP sample to the Ct for the pre-IP sample, normalized to the value obtained from the PBS-injected mice (Mean ± SEM; n = 3; *, p<0.05; **, p<0.01; student's t-test). (D) The experiment from (C) was repeated with the modification that all mice were pre-injected with Ro-32–0432, a systemic PKC inhibitor (Mean ± SEM; n = 3; student's t-test).

We immunoprecipitated mRNP complexes from lysates prepared from mouse forebrain using an antibody that recognizes all 3 nELAV RBPs. Real-time quantitative PCR analysis of the co-precipitated mRNAs was used to identify whether specific *Bdnf* mRNA isoforms were associated with the immunoprecipitated mRNPs, using *Rpl10A* mRNA for normalizing between samples, as it has been previously shown not to interact with nELAV proteins [22]. Primers for *Bdnf* mRNA isoforms I, IIc, IV and VI were used, as these species are found in relative abundance in adult mouse brain. ΔCt values were found to be significantly lower for mRNP IPs using the pan-nELAV antibody as compared to those using the mouse IgG isotype control, corresponding to a 12.4-, 16.9-, 13.3- and 17.3-fold change, respectively (Fig. 1A), demonstrating an *in vivo* association of nELAV RPBs with all 4 *Bdnf* mRNA isoforms examined.

It has been previously demonstrated that BDNF expression can be regulated by neuronal activity[24,27], so we decided to test whether the association of nELAV proteins with *Bdnf* mRNAs could be regulated in a similar way, as might be expected if this interaction is important in mediating activity-dependent changes in BDNF expression. We used intraperitoneal (IP) injection of seizure-inducing doses of pilocarpine to elevate neuronal activity and prepared forebrain lysate 30 minutes post-injection for use in mRNP immunoprecipitations as described above (Fig. 1B). At 30 minutes post pilocarpine injection, the abundance of *Bdnf* mRNA is not changed [13], which would simplify the interpretation of the data from mRNP immunoprecipitation. Ct values for samples from pan-nELAV antibody immunoprecipitations were normalized to Ct values for the corresponding total mRNP sample (pre-immunoprecipitation), and found to be lower when mice were injected with pilocarpine as compared to negative control mice injected with PBS, resulting in a 1.8-, 2.0-, 2.0- and 2.2-fold change in percent recovery of *Bdnf* mRNA isoforms I, IIc, IV and VI, respectively (Fig. 1C). Since protein kinase C (PKC) has been shown to phosphorylate nELAV RBPs to regulate their trafficking and mRNA stabilizing properties [18], we repeated the pilocarpine stimulation experiment in mice pre-treated with Ro-32–0432, a systemic inhibitor of protein kinase C. This resulted in failure of pilocarpine stimulation to increase the recovery of *Bdnf* mRNA-containing mRNPs (Fig. 1D), implicating PKC activity as an important mediator of this effect.

### *Bdnf* 5' UTR sequences negatively regulate mRNA translation

**Figure 2:**
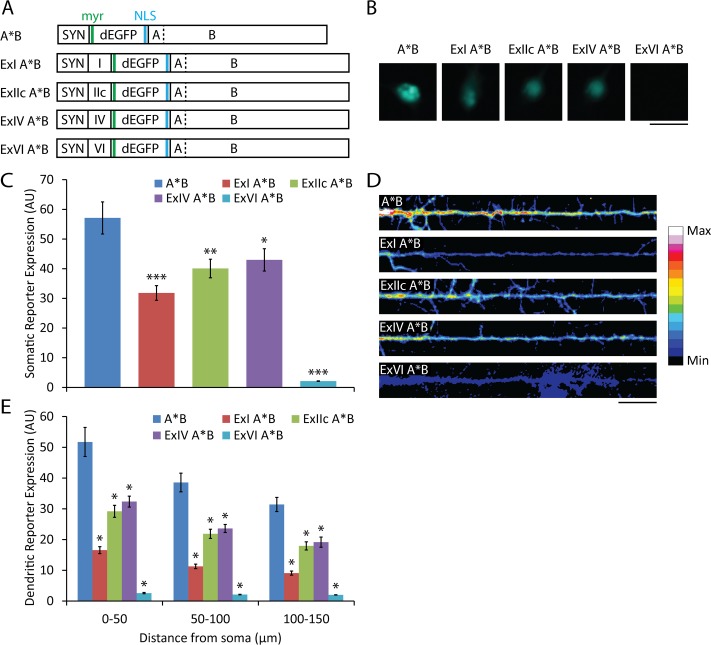
Effect of *Bdnf* 5' UTR sequences on local reporter synthesis. (A) Schematic representing the constructs used. SYN, human synapsin promoter; I, IIc, IV, VI, *Bdnf* 5' UTR sequences; dEGFP, destabilized EGFP; myr, myristoylation sequence; NLS, nuclear localization sequence; A, short *Bdnf* 3' UTR; B, long *Bdnf* 3' UTR; dashed line, non-functional mutated polyadenylation site. (B) Somatic GFP fluorescence from dissociated rat hippocampal cultures transfected at 7 DIV with the indicated constructs and fixed and imaged at 9 DIV (scale bar, 20 μm). (C) Mean ± SEM from images represented in (B) (n = 30; *, p<0.05; **, p<0.01; ***, p<0.001; student's t-test). Intensity of GFP fluorescence is shown in arbitrary unit (AU). (D) Colorized dendritic GFP fluorescence from hippocampal cultures transfected at 7 DIV with the indicated constructs and fixed and imaged at 9 DIV, using min and max pixel values from representative images across all conditions to generate the color table (scale bar, 20 μm). (E) Mean ± SEM in 50-μm bins from images represented in (D) (n = 20; *, p<0.001; student's t-test).

To establish an *in vitro* system to study the effects of nELAV RBPs on the regulation of mRNAs containing *Bdnf* sequences, we decided to include both 5'- and 3'-UTR sequences in a reporter construct that expresses destabilized GFP modified to include a myristoylation peptide and a nuclear localization sequence (NLS), restricting movement of GFP signal distal to the site of synthesis and serving as a more accurate indicator of local translation (Fig. 2A).

Inclusion of the 5' UTRs of *Bdnf* mRNA isoforms I, IIc, IV and VI reduced expression of a local translation reporter containing the long *Bdnf* 3' UTR with the first polyadenylation site mutated (A*B) so that only the long 3' UTR species is generated (Fig. 2). The pattern of reduced expression of the local translation reporter was consistent between somatic and dendritic compartments (Fig. 2B-E), suggesting decreased translation or stability of the mRNAs containing the different *Bdnf* 5' UTRs. The 5' UTR from *Bdnf* mRNAs transcribed from promoter VI resulted in the largest decreases in somatic and dendritic reporter expression, 96% and 95%, respectively, while the 5' UTR from *Bdnf* mRNAs transcribed from promoter IV resulted in the smallest somatic and dendritic decreases, 25% and 39%. To potentially minimize confounds resulting from negative regulation of local translation acting on 5' UTR sequences we used the *Bdnf* 5' UTR from isoform IV in all further local translation reporter constructs.

### PKC activation increases dendritic translation of mRNAs with the long *Bdnf* 3' UTR in an HuD-dependent manner

It has previously been demonstrated that neuronal activity can increase local translation of *Bdnf* mRNAs in dendrites. This has been shown in hippocampal culture using chemical LTP induction through tetraethylammonium (TEA) treatment [13]. In order to gain more insight into the interaction between neuronal Hu RBPs and *Bdnf* mRNAs we investigated the role of PKC activation in local translation. The PKC pathway is known to be activated by TEA induction of LTP [28], and is important for the transport of nELAV RBPs to their sites of action [18], and thus may play a role in mediating any effects they may have on regulating local translation of *Bdnf* mRNAs.

**Figure 3:**
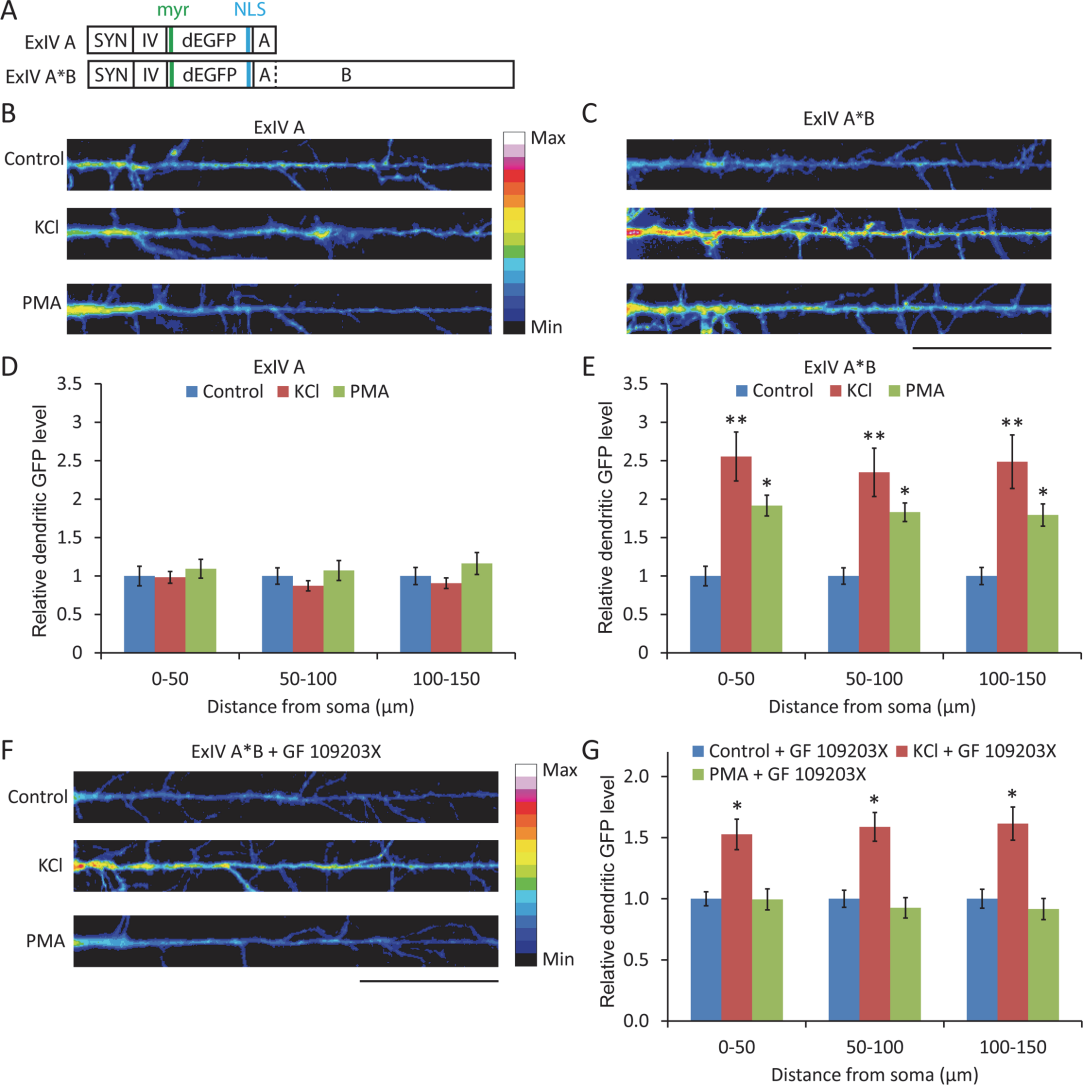
PMA treatment leads to PKC-dependent increases in dendritic translation of local reporter constructs with the long *Bdnf* 3' UTR. (A) Schematic representing the constructs used. (B and C) Colorized dendritic GFP fluorescence from dissociated rat hippocampal cultures transfected at 14 DIV with the indicated constructs and on 16 DIV treated for 1 hour with KCl, PMA or vehicle, fixed and then imaged (scale bar, 20 μm). (D and E) Mean ± SEM in 50-μm bins from images represented in (B) and (C) (n = 25; *, p<0.05; **, p<0.01; student's t-test). (F) Colorized dendritic GFP fluorescence from hippocampal cultures transfected at 14 DIV with ExIV A*B and on 16 DIV pre-treated for 30 min with GF109203X, a PKC inhibitor, then treated for 1 hour with KCl or PMA, fixed and then imaged (scale bar, 20 μm). (G) Mean ± SEM in 50-μm bins from images represented in (F) (n = 25; *, p<0.001; student's t-test).

Rat dissociated hippocampal cultures were transfected at 14 DIV with local translation reporter constructs containing either the short (ExIV A) or long (ExIV A*B) *Bdnf* 3' UTRs (Fig. 3A) and treated 2 days later for 1 hour with 30 mM KCl or 100 nM PMA. Neurons transfected with ExIV A*B, but not ExIV A, showed significant increases in local dendritic translation compared to controls for both treatments (Fig. 3B-E). Furthermore, the effect of PMA treatment was completely abrogated in cultures pre-treated with the PKC inhibitor GF 109203X, whereas the effect of KCl stimulation was only partially reduced (Fig. 3F, G), suggesting other pathways besides PKC are involved in depolarization-dependent regulation of mRNAs with the A*B sequence.

**Figure 4:**
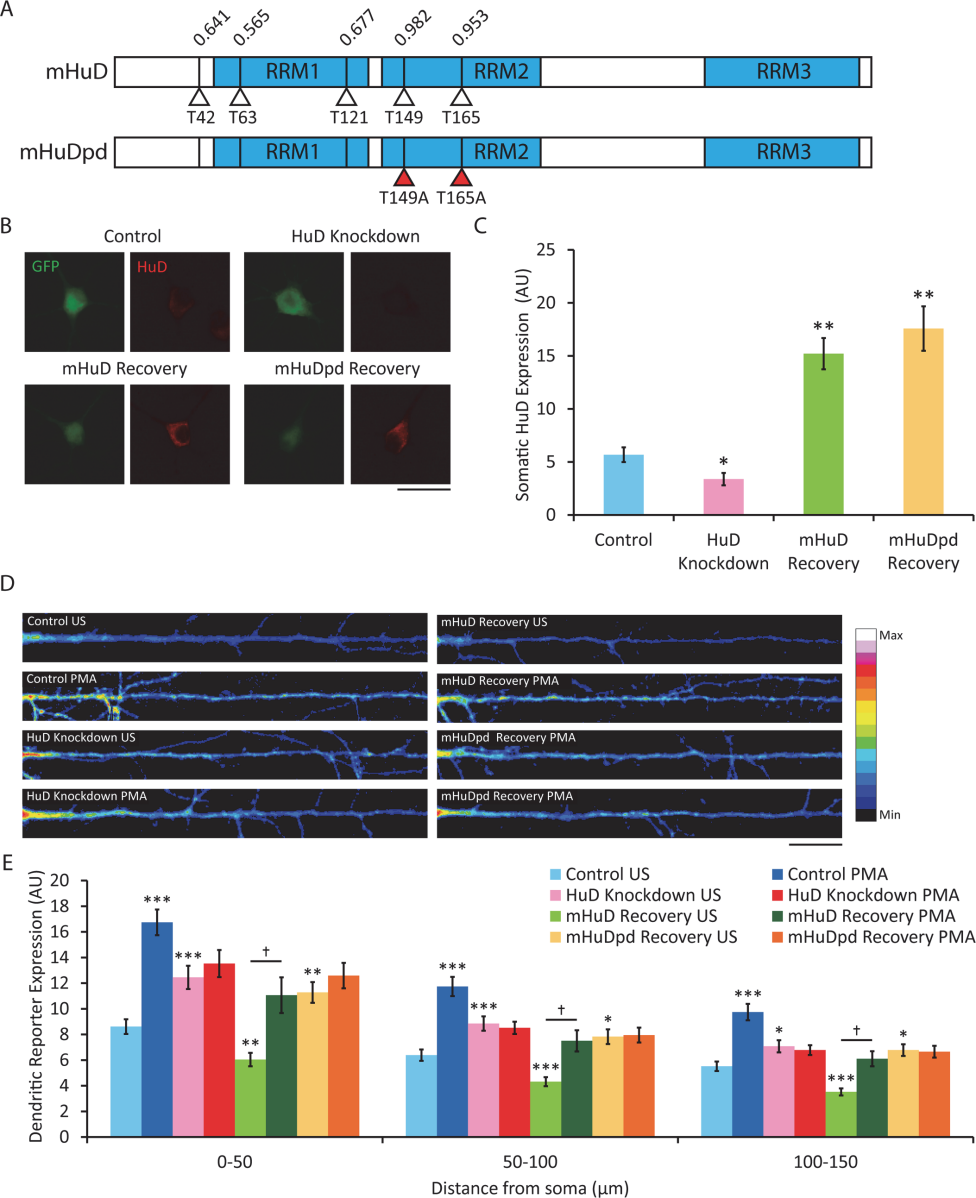
PKC-dependent increases in dendritic translation of local reporter constructs with the long *Bdnf* 3' UTR are mediated by HuD. (A) Schematic representing the mouse HuD overexpression cassettes with *in silico*-predicted threonine phosphorylation sites and their scores (RRM1–3, RNA recognition motifs; open triangles, predicted threonine phosphorylation sites; closed triangles, threonine-to-alanine substitutions). (B) Immunocytochemistry of hippocampal cultures transfected with the indicated plasmids on 14 DIV, then fixed, stained with monoclonal anti-HuD antibody and imaged on 16 DIV (Control: ExIV A*B, pSuper, pcDNA; HuD Knockdown: ExIV A*B, rHuD shRNA, pcDNA; mHuD Recovery: ExIV A*B, rHuD shRNA, mHuD; mHuDpd Recovery: ExIV A*B, rHuD shRNA, mHuDpd; scale bar, 20 μm). (C) Mean ± SEM of soma from images represented in (B) (n = 30; *, p<0.05; **, p<0.001; student's t-test). (D) Colorized dendritic GFP fluorescence from hippocampal cultures transfected at 14 DIV with the indicated plasmids, then on 16 DIV treated for 1 hour with PMA or vehicle, fixed and then imaged (scale bar, 20 μm). (E) Mean ± SEM in 50-μm bins from images represented in (D) (n = 40; *, p<0.05; **, p<0.01; ***, p<0.001; student's t-test against control for each bin; †, p<0.001; student's t-test against conditions underneath bar).

HuD, one of the more well-studied members of the nELAV RBP family, has been found to be upregulated in rat hippocampus after spatial learning, concomitant with increases in association with and stability of target mRNA, leading to increased translation [16,29]. We investigated the importance of HuD in the PKC-mediated upregulation of the local reporter construct detailed above by co-transfecting hippocampal cultures with a plasmid encoding an shRNA specific to rat *HuD* mRNA. HuD protein levels as detected by immunocytochemistry using a monoclonal antibody against HuD were decreased by 40% in the soma of co-transfected neurons (Fig. 4B, C), though there may be some cross-reactivity with the other nELAV proteins, in which case the actual reduction in HuD would be even greater than estimated. In cultures co-expressing the local synthesis reporter construct and the rat HuD shRNA, PMA treatment failed to increase dendritic reporter expression (Fig. 4D, E), suggesting an important role for HuD in mediating this effect. Interestingly, the basal level of dendritic reporter expression was increased compared to controls with normal HuD protein levels. Recovery experiments were conducted by co-transfecting the reporter and shRNA constructs with plasmids overexpressing either wild-type mouse HuD (mHuD) or a putative phosphorylation-deficient mutant (mHuDpd). *In silico* prediction of threonine phosphorylation sites in mouse HuD revealed 5 candidate residues, 2 with very high confidence scores, Thr149 and Thr165, which were substituted with alanine residues to generate mHuDpd (Fig. 4A). Overexpression of mHuD or mHuDpd in the presence of the shRNA lead to an increase in somatic HuD levels to 268% or 309% of control, respectively. Recovery with mHuD, but not with mHuDpd, restored the PKC-mediated increase in dendritic synthesis of the local reporter (Fig. 4D, E), indicating that the effect of the shRNA is specifically due to reduced HuD, and that one or both of the substituted threonine residues is a phosphorylation site of PKC necessary for mediating the ability of HuD to increase translation of its target mRNA. Recovery with mHuD resulted in a decrease in basal dendritic translation of the local reporter, while recovery with mHuDpd resulted in levels that were no different than those from the knockdown of HuD alone. These findings suggest that non-phosphorylated HuD inhibits translation of its target mRNAs, and this repression is relieved by PKC phosphorylation.

### HuD can directly regulate local protein synthesis

**Figure 5:**
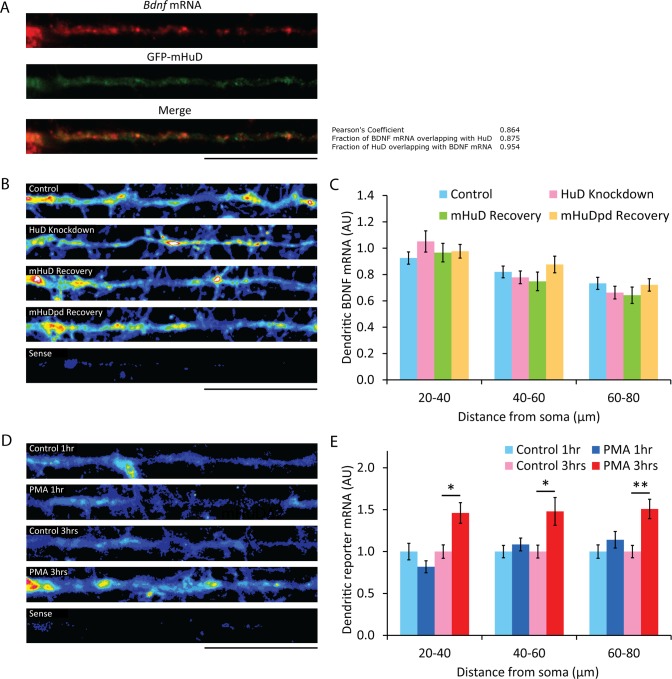
Changes in HuD expression and short-term PMA treatment do not affect levels of dendritic mRNAs with *Bdnf* 5' and 3'-UTRs. (A) Dual *Bdnf* mRNA FISH and HuD immunocytochemistry on hippocampal cultures transfected with recombinant GFP-mHuD at 14 DIV and fixed, stained and imaged at 16 DIV, using antisense probe against the *Bdnf* coding sequence for *in situ* and anti-GFP for immunocytochemistry (n = 30; scale bar, 20 μm). Co-localization data was obtained using the JACoP plugin on ImageJ. (B) Colorized dendritic *in situ* signal using a probe against the *Bdnf* mRNA coding sequence in hippocampal cultures transfected with the indicated plasmids at 14 DIV and then fixed at 16 DIV (sense, sense *in situ* probe; scale bar, 20 μm). (C) Mean ± SEM in 20-μm bins from images represented in (B), normalized to the mean dendritic signal from 20–40 μm away from the soma of untransfected neurons on the same coverslip (n = 30; *, p<0.05; **, p<0.01; student's t-test). (D) Colorized dendritic *in situ* signal using a probe against the GFP coding sequence in hippocampal cultures transfected with the reporter construct ExIV A*B at 14 DIV and then treated for 1 or 3 hours with PMA or vehicle, then fixed at 16 DIV (sense, sense *in situ* probe; scale bar, 20 μm). (E) Mean ± SEM in 20-μm bins from images represented in (D) (n = 30; *, p<0.01; **, p<0.001; student's t-test).

If HuD interacts with *Bdnf* mRNAs to regulate their dendritic translation, they should co-localize in these compartments. Hippocampal cultures were transfected to express a GFP-mHuD fusion protein, and co-localization of this protein with *Bdnf* mRNA was assessed using dual fluorescent *in situ* hybridization (FISH) and immunocytochemistry. Intensity correlation coefficient-based (ICCB) analysis yielded a pearson's coefficient of 0.864, with 95% of the GFP-mHuD signal overlapping with *Bdnf* mRNA (Fig. 5A), suggesting a high degree of dendritic association even in the absence of stimulation. If these mRNP complexes of *Bdnf* mRNA with HuD represent RNA transport granules, HuD could be involved in regulating target mRNA trafficking to dendrites. Thus altering HuD levels through shRNA knockdown and heterologous overexpression would affect the dendritic localization of target mRNAs, potentially resulting in the observed differences in basal dendritic expression of the local reporter under decreased or elevated HuD levels. However, FISH with a probe directed against the *Bdnf* coding region showed no difference in *Bdnf* mRNA levels in the dendrites of hippocampal neurons after HuD knockdown or recovery (Fig. 5B, C), indicating that HuD is not essential for the dendritic trafficking of *Bdnf* mRNAs.

Increased neuronal activation has been shown to increase *Bdnf* mRNA trafficking to dendrites [30], and it is possible that PKC activation induced by PMA treatment could result in the observed increase in dendritic reporter synthesis as a consequence of an increased pool of dendritic reporter mRNAs, rather than by directly affecting local translation of pre-existing dendritic mRNAs. To distinguish between these two mechanisms we performed FISH on control or PMA-treated cultures transfected with the local reporter, using probes directed against the GFP coding sequence. After 1 hour of PMA treatment, the length of time used in previous experiments to assess local reporter synthesis, there was no detectable increase in dendritic reporter mRNA levels (Fig. 5D, E), indicating the increased local reporter synthesis after this treatment is a result of increased translation of pre-existing mRNAs. However, 3 hours of PMA treatment led to significantly increased dendritic reporter mRNA levels, suggesting a complex role for PKC in regulating dendritic translation of mRNAs with the *Bdnf* long 3' UTR, mediating both rapid, short-term increases by acting directly on local translation and slower, long-term increases through increased trafficking of mRNAs to sites of activity.

### HuD dendritic localization requires threonine phosphorylation

**Figure 6:**
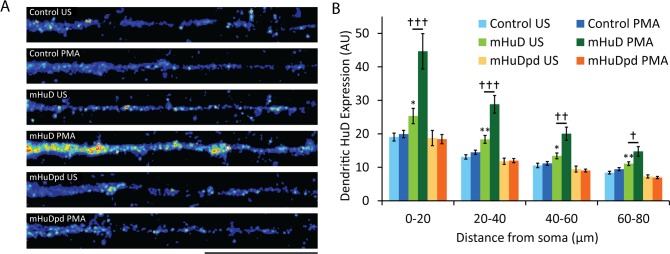
HuD transport to dendrites requires phosphorylation at T149 and/or T165. (A) Colorized dendritic HuD immunocytochemistry signal in hippocampal cultures transfected at 14 DIV with the indicated plasmids, then treated for 1 hour with PMA or vehicle, fixed, stained with monoclonal anti-HuD antibody and imaged at 16 DIV (US, unstimulated; scale bar, 20μm). (B) Mean ± SEM in 20-μm bins from images represented in (A) (n = 30; *, p<0.05; **, p<0.01; student's t-test against control for each bin; †, p<0.05; student's t-test against conditions underneath bar).

The trafficking of HuD has been reported to depend on its phosphorylation by PKC [18]. Immunocytochemistry with a monoclonal antibody directed against HuD revealed an increase in dendritically-localized HuD after overexpression of mHuD, but not mHuDpd (Fig. 6A, B), which is consistent with the observed changes in basal dendritic translation of the local reporter if HuD is a repressor of translation under resting conditions. Dendritic levels of HuD were further increased after PMA treatment only in neurons overexpressing mHuD (Fig. 6A, B), further demonstrating the likelihood that one or both of the substituted threonine residues in mHuDpd is phosphorylated by PKC to regulate its translocation to dendrites and relieve repression of its target mRNAs.

### HuD interacts directly with *Bdnf* mRNA sequences

**Figure 7:**
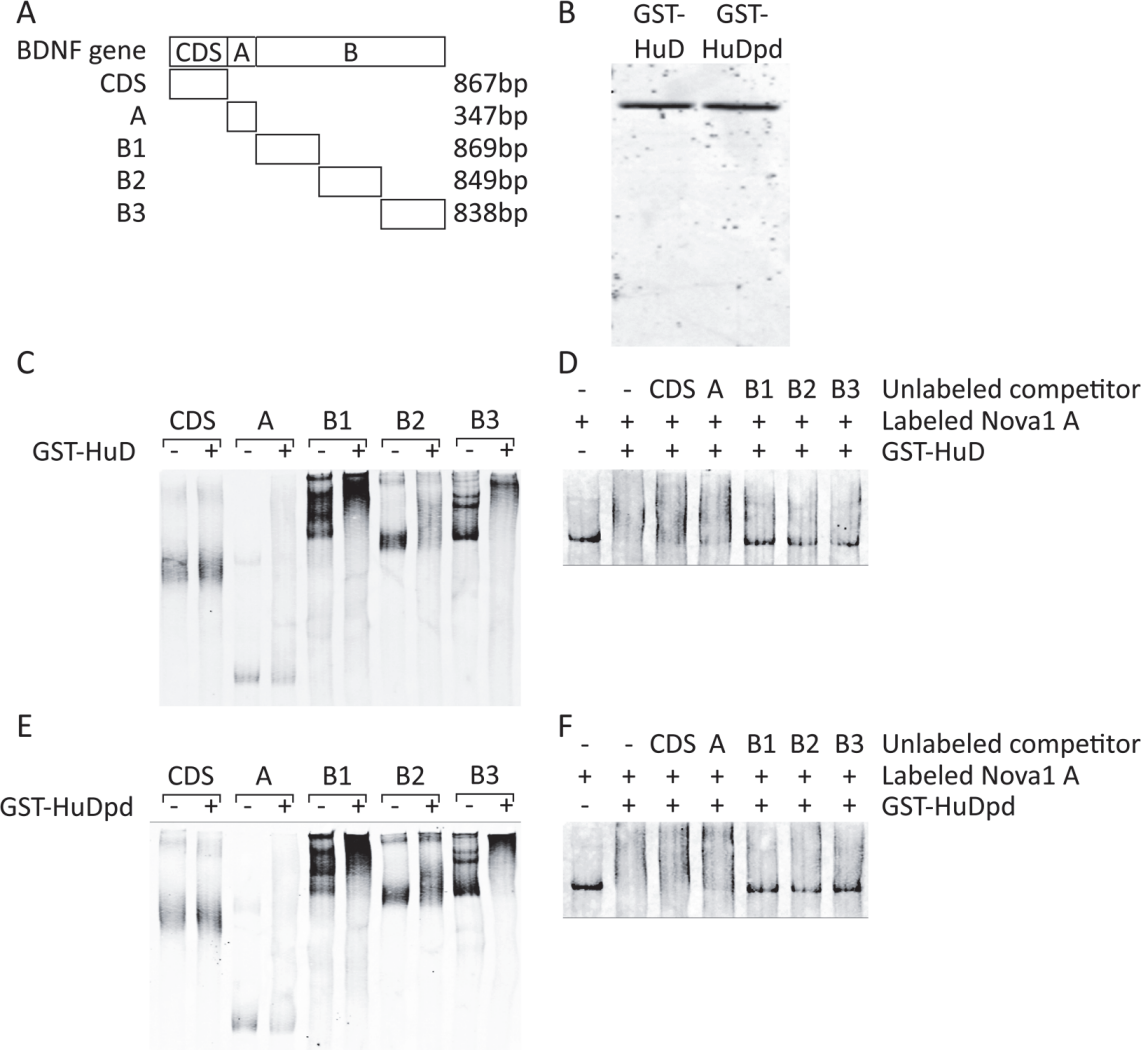
HuD directly interacts with sequences in the long *Bdnf* 3' UTR, and this interaction is not impaired by substitution of PKC phosphorylation sites important for upregulation of its target mRNAs. (A) Schematic showing the regions of the *Bdnf* gene covered by the RNA probes used in the REMSA assay. (B) SDS PAGE of the purified recombinant GST-HuD and GST-HuDpd, stained with coomassie brilliant blue to show the extent of enrichment of the overexpressed GST fusion products. (C and E) Biotin-labeled RNA probes corresponding to the *Bdnf* mRNA regions shown in (A) were incubated with buffer (-) or the indicated purified recombinant GST fusion protein (+) and visualized on non-denaturing PAGE using IRdye-conjugated streptavidin. (D and F) Biotin-labeled RNA probe corresponding to the positive control *Nova1* 3' UTR sequence was incubated with buffer, the indicated GST fusion protein alone, or the protein pre-incubated with unlabeled competitor RNAs covering the sequences shown in (A) and visualized as in (C) and (E).

mRNP complexes can be composed of multiple proteins and distinct mRNAs, and while the previous data has indicated an association of HuD with *Bdnf* mRNAs [19], whether HuD interacts with the long *Bdnf* 3' UTR is unknown. REMSAs were carried out with recombinant GST-HuD or GST-HuDpd and labeled RNA probes corresponding to the coding sequence and 3' UTR of *Bdnf* (Fig. 7A, B). All of the probes corresponding to long *Bdnf* 3' UTR sequences, B1, B2 and B3, showed an upward shift in the presence of GST-HuD or GST-HuDpd, with the greatest effect observed for probes B1 and B3, while the *Bdnf* coding sequence probe, CDS, and the short 3' UTR probe, A, showed little or no shift (Fig. 7C, E). To complement these results we performed competition REMSAs, adding a labeled probe corresponding to the *Nova1* 3' UTR, a known target of HuD [22], to GST-HuD or GST-HuDpd that was pre-incubated with unlabeled *Bdnf* probes. Only the long *Bdnf* 3' UTR probes were able to compete for binding with the recombinant proteins and prevent an upward shift of the labeled *Nova1* 3' UTR probe (Fig. 7D, F). These findings demonstrate a direct interaction between HuD and sequences in the long *Bdnf* 3' UTR, and further show that substitution of the two threonine residues with alanines has no effect on the mutant protein's ability to interact with its target mRNAs, validating its use in earlier experiments to determine the relevance of the two residues as potential PKC phosphorylation sites.

## Discussion

### *Bdnf* mRNAs interact directly with HuD

The neuronal Hu proteins are likely candidates for being *trans*-acting factors regulating the stability and translation of *Bdnf* mRNAs, based on the presence of HuD-binding motifs in the long *Bdnf* 3' UTR [31], association of HuD with *Bdnf* mRNA in cultured hippocampal neurons [19], and related functional consequences arising from perturbations in neuronal Hu protein levels or *Bdnf* mRNA expression [32,33]. We performed quantitative RT-PCR analysis on RNAs co-immunoprecipitated from mouse forebrain lysate with a pan neuronal Hu antibody and found enrichment of all *Bdnf* mRNA isoforms examined, indicating their *in vivo* association with neuronal Hu proteins.

We next sought to uncover whether this association could be modulated by neuronal activation, as might be expected if HuD were involved in mediating activity-dependent changes in BDNF expression. We repeated the quantitative RT-PCR experiments using tissue from animals with elevated neuronal activity as a result of seizures induced by pilocarpine injection, in the presence or absence of a systemic inhibitor of PKC. Elevated neuronal activation significantly increased the percent of *Bdnf* mRNAs co-immunoprecipitated as a fraction of input RNA compared to animals with basal neuronal activity, and systemic inhibition of PKC completely reversed this increase, indicating the activity-dependent modulation depends on PKC phosphorylation. This increased co-immunoprecipitation could result from increased formation of mRNP complexes containing neuronal Hu proteins and *Bdnf* mRNAs, or the stabilization of existing complexes following HuD phosphorylation by PKC.

To determine whether there is a direct interaction between neuronal Hu proteins and *Bdnf* mRNA we performed REMSAs using purified recombinant HuD and labeled RNA probes corresponding to sequences in the coding region and 3' UTR of *Bdnf*. We also performed competition assays using unlabeled *Bdnf* probes and a labeled probe corresponding to a sequence in the *Nova1* 3' UTR previously shown to interact with HuD [22]. Only probes corresponding to sequences in the long *Bdnf* 3' UTR were found to interact strongly with HuD in both assays, indicating the presence of multiple HuD binding sites throughout this region. These findings do not contradict with the observation that HuD interacts with the short *Bdnf* 3' UTR [19], as we also observed this interaction, although it is much weaker than the interaction between HuD and the B segment of the long *Bdnf* 3' UTR.

The physiological relevance of these results is highlighted by the similar subcellular distributions of *Bdnf* mRNAs and HuD, with both found either in translationally repressed mRNP complexes or associated with polysomes, sites of active translation [13,34]. Activation of PKC results in the nucleocytoplasmic shuttling of Hu proteins, their threonine phosphorylation, and increased association of the cytoskeleton and polysomes [18], establishing a pathway whereby neuronal activity could lead to HuD-mediated increase in BDNF synthesis.

### *Bdnf* 5' UTRs repress translational efficiency

A recent study found that 5' UTRs play an important role in regulating translation of *Bdnf* mRNAs in SH-SY5Y cell line [35]. Our study indicates that 5' UTRs also control translation of *Bdnf* mRNA in cultured primary neurons. We found that including 5' UTRs from *Bdnf* isoforms I, IIc, IV and VI in a local reporter construct decreased both somatic and dendritic reporter expression, with the relative changes being similar between the two compartments. The observed translational repressive effect of exons I, IIc, IV and VI is in agreement with the recently published analysis of rat 5' UTR sequences on translatability of *Bdnf* mRNA [35]. The isoform IV 5' UTR conferred the least reduction in translation, while the isoform VI 5' UTR almost completely blocked expression. These 2 *Bdnf* isoforms are among the most abundant in adult rat hippocampus [13,36], with isoform IV expression being more activity-dependent than that of isoform VI [24,37]. It has also been reported that isoform VI, but not isoform IV, is targeted to dendrites after neuronal activation [38]. It seems contradictory that the *Bdnf* mRNA isoform least increased by activity would be most localized to distal dendrites, where activity-dependent local translation could be an important process leading to synaptic remodeling.

### PKC activation can increases local dendritic translation of RNAs with the long *Bdnf* 3' UTR

Expression of a local reporter with the long, but not the short, *Bdnf* 3' UTR was increased in the distal dendrites of hippocampal neurons stimulated either by KCl depolarization or PMA-mediated PKC activation. This result is in agreement with a previous finding that chemical LTP induction through TEA treatment can selectively increase dendritic expression of a local translation reporter with the long, but not the short, *Bdnf* 3' UTR [13]. Pre-treatment of cultures with an inhibitor of PKC completely abolished the increased dendritic translation after PMA treatment, as expected if PMA is acting through PKC, and was able to reduce, but not block, the increased reporter synthesis after KCl stimulation, suggesting that depolarization can trigger PKC-dependent and—independent pathways to increase translation of mRNAs with the long *Bdnf* 3' UTR. Dendritic reporter translation could be modulated either by a change in the pool of dendritic reporter mRNAs or by a direct effect on the rate of translation of pre-existing dendritic mRNAs. While we found that 3 hours of PMA treatment was able to increase reporter mRNA levels in dendrites, the 1 hour treatment used in reporter assay experiments did not result in any significant difference, suggesting the PKC activity-dependent increase in reporter expression is due to a mechanism operating to increase the translation of pre-existing local mRNAs.

The regulation of BDNF synthesis in dendrites by PKC may be an important pathway involved in learning and memory. PKC activity has been shown to be increased during both the induction and maintenance phases of LTP at Schaffer collateral-CA1 synapses [39], and the constitutively active, atypical PKC isozyme, PKM(, is necessary and sufficient for the maintenance phase of LTP [40]. Since LTP induction does not require protein synthesis, the relevance of PKC-mediated increases in dendritic BDNF translation is likely during LTP maintenance. PKM (activity during this period could increase BDNF synthesis in stimulated spines, leading to enhanced TrkB activation and downstream signaling through Akt-mTOR and Ras-ERK pathways to remodel the spine and increase synaptic strength. Despite the lack of a requirement for protein synthesis during the induction phase of LTP, synthesis of BDNF in activated spines during this period may be physiologically relevant to prime these spines with additional stores of BDNF to facilitate the later, protein synthesis-dependent maintenance phase of LTP.

### PKC activity can relieve HuD-mediated translational repression of mRNAs with the long *Bdnf* 3' UTR

Knocking down endogenous HuD in rat hippocampal cultures blocked the ability of PKC activation to increase dendritic translation of mRNAs with the long *Bdnf* 3' UTR. Unexpectedly, this also resulted in a significant increase in dendritic expression of the local reporter in unstimulated cultures. Rescue with wild-type mouse HuD increased dendritic HuD above control levels and restored the response of the local reporter to PKC activation, while decreasing its basal dendritic translation. These data are consistent with HuD either restricting dendritic trafficking of mRNAs with the long *Bdnf* 3' UTR or inhibiting their translation under basal conditions. Rescue with a putative phosphorylation-deficient mutant of HuD failed, resulting in elevated basal reporter expression that is insensitive to PKC activation, similar to HuD knockdown alone. One or both of the threonine residues substituted in the mutant are likely PKC phosphorylation sites, as PKC activation was able to increase dendritic localization of the overexpressed wild-type HuD, but not the mutant. Its failure to decrease basal dendritic reporter translation, despite retaining its ability to bind *Bdnf* mRNA sequences and its high somatic expression, argues against a role for HuD in regulating dendritic mRNA trafficking. Furthermore, we did not observe any changes in the amount of *Bdnf* mRNA in the dendrites of hippocampal neurons after knockdown or overexpression of HuD.

**Figure 8:**
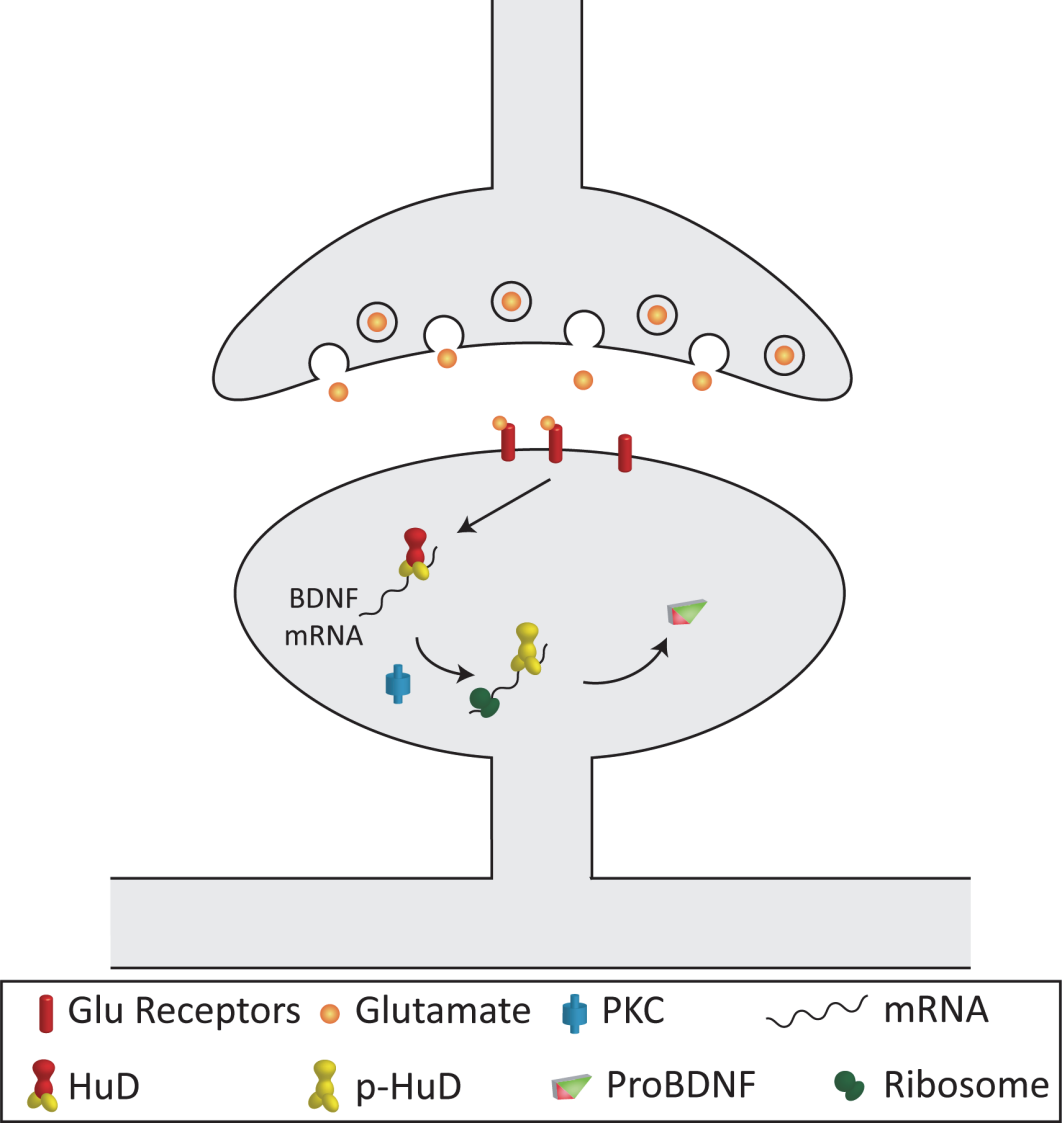
A model on *Bdnf* mRNA translation. The model illustrates the proposed mechanism of the PKC-mediated component of activity-dependent upregulation of local *Bdnf* mRNA translation in dendrites.

Our data suggests a model where HuD can repress translation of mRNAs with the long *Bdnf* 3' UTR, and PKC activation can relieve this repression, possibly by direct phosphorylation of HuD itself (Fig. 8). While many reports on HuD find that it increases translation of its target mRNAs [22,41,42], it has also been found to inhibit translation of p27 [43] and tau [44], and 2 of its RNA binding sites have been identified as the most overrepresented motifs in the 3' UTRs of mRNAs downregulated after expression of miRNAs, and in the 3' UTRs of mRNAs bound by Argonaute [45], an integral component of P-bodies, sites of translational repression [46]. Thus HuD could be an inhibitor of translation in certain contexts, such as for genes regulated by miRNA inhibition, like *Bdnf* [47,48]. Notably, the long *Bdnf* 3' UTR has been found to repress basal translation [13], and our results indicate HuD has multiple binding sites throughout this region. Further studies are necessary to demonstrate whether the absence of HuD binding can increase the proportion of *Bdnf* mRNAs associated with polysomes, and if PKC phosphorylation on either of the two threonine residues mutated in this study is the signal leading to polysome association.

Neuronal activity should regulate dendritic BDNF synthesis through other signaling pathways in addition to the PKC-HuD pathway, because we found that a PKC inhibitor was able to reduce, but not block, the increased dendritic synthesis of a reporter with the long *Bdnf* 3' UTR after KCl-induced depolarization. Activation of the NMDA receptor has been shown to stimulate dendritic synthesis of Ca^2+^/calmodulin-dependent protein kinase II α subunit through phosphorylation of translation elongation factor eEF2 or cytoplasmic-polyadenylation-element-binding protein [49,50]. It would be interesting to investigate whether these signaling pathways also regulate dendritic BDNF synthesis in future studies.
